# Prevalence of Underweight, Overweight and Obesity in School-Aged Children in the Urban Area of the Northwestern Part of Romania

**DOI:** 10.3390/ijerph18105176

**Published:** 2021-05-13

**Authors:** Tudor Lucian Pop, Dana Maniu, Daniela Rajka, Cecilia Lazea, Gabriel Cismaru, Adrian Ştef, Simona Sorana Căinap

**Affiliations:** 1 2nd Pediatric Discipline, Mother and Child Department, “Iuliu Hatieganu” University of Medicine and Pharmacy, 400012 Cluj-Napoca, Romania; cainap.simona@gmail.com; 2 2nd Pediatric Clinic, Emergency Clinical Hospital for Children, 400177 Cluj-Napoca, Romania; 3 Romanian Society of Social Pediatrics, 400177 Cluj-Napoca, Romania; 4 Faculty of Physics, Babes-Bolyai University, 400084 Cluj-Napoca, Romania; dana.maniu@phys.ubbcluj.ro; 5 Society of Physicians from Children and Youth Communities, 400427 Cluj-Napoca, Romania; rajkadani07@yahoo.com; 6 1st Pediatric Discipline, Mother and Child Department, “Iuliu Hatieganu” University of Medicine and Pharmacy, 40012 Cluj-Napoca, Romania; 7 1st Pediatric Clinic, Emergency Clinical Hospital for Children, 400470 Cluj-Napoca, Romania; 8 Cardiology-Rehabilitation Discipline, Internal Medicine Department, “Iuliu Hatieganu” University of Medicine and Pharmacy, 400012 Cluj-Napoca, Romania; cismaru.gabriel@umfcluj.ro; 9 Cardiology Department, Rehabilitation Hospital, 400347 Cluj-Napoca, Romania; 10 Department of Surgery, “Iuliu Hatieganu” University of Medicine and Pharmacy, 400012 Cluj-Napoca, Romania; stef.adrian@yahoo.com; 11 “Nicolae Stancioiu” Heart Institute, 400001 Cluj-Napoca, Romania

**Keywords:** overweight, obesity, underweight, children, adolescents, urban area

## Abstract

Introduction: During the last three decades, there has been an excess weight epidemic due to changes in nutrition and lifestyle. Few data on the prevalence of overweight and obesity in children in Romania were published, without a single study representative at the national level. There are reports on the higher level of overweight and obesity in urban areas compared to rural ones. This study aimed to estimate the prevalence of underweight, overweight, obesity and severe obesity in children enrolled in schools from the urban area. Material and methods: For this cross-sectional study, children from 177 schools from the urban area of five counties from the northwestern part of Romania were included after the parents signed written informed consent. Anthropometric data were recorded (weight, height) based on World Health Organization (WHO) recommendations and Body-Mass-Index (BMI), and the z-score for BMI were calculated. The nutritional status was estimated using three reference criteria: WHO, International Obesity Task Force (IOTC) and the Center for Disease Control and Prevention (CDC). Results: We analyzed data of 21,650 children (48.19% boys) age between 7 and 18 years. The prevalence of overweight was 13.8%, 16.2% and 20.3%, of obesity was 10.7%, 10.0% and 5.7% and of severe obesity was 5.1%, 1.2% and 1.6% (using WHO, CDC and IOTF cut-offs). Underweight was present in 5.2% (WHO), 6% (CDC) and 2.6% (IOTF). The highest prevalence of overweight (including obesity) was found in children aged 10 years, and the lowest in adolescents at 18 years. Boys have a higher prevalence of excess weight than girls. Using IOTF cut-offs, the prevalence of obesity and severe obesity was lower than using WHO criteria. Conclusions: The prevalence of overweight (including obesity) in children from the urban area of Western Romania was recorded at alarming levels, higher in boys and at the pre-puberty ages. There are significant differences based on the reference system used. It is important to correctly choose the reference for the definition of overweight and obesity to have the correct estimation of the target for public health measures.

## 1. Introduction

Childhood obesity is a significant public health problem all over the World [1,2]. Globally, the prevalence of obesity and overweight was tripled in children and adolescents from 1975 (4%) till 2016 (over 18%). In 2016, more than 330 million children and adolescents were overweight or obese [3]. There are estimations that in 2030, almost 30% of all children will be overweight and obese [4]. The increase was higher until 2000 (as in adults), followed by a plateau, mainly in developed countries. In low-income and middle-income countries, there is a transition from underweight to overweight and obesity [3,5,6]. Across the World, there is an increased heterogeneity in the prevalence of obesity with implications for global actions and targets [3]. World Health Organization (WHO) estimates that most children with overweight live in undeveloped countries where the rate of increase in the prevalence of obesity is higher than in developed countries [1,5,7]. 

Compared with overweight and obesity, underweight received less attention in European studies, with few comprehensive studies [8]. In 2016 the prevalence of underweight was estimated at 10.4% in Europe [6]. There is wide variability of underweight prevalence in European countries, with estimations decreasing from 18.1% in Hungarian children aged 2–9 years and 16% in girls aged 11 years in Wales to 1% in adolescents from Slovenia, Finland or Norway [8,9]. 

Obesity has a multifactorial origin, involving genetic and environmental factors [10]. Overweight and obesity are associated with lifestyle factors such as less frequent physical activity and increased sedentary activity (computer games, internet use, television), less consumption of breakfast, vegetables and regular sugar-sweetened beverages or fast-food consumption [11,12]. The diet change, consuming food high in carbohydrate and fat content, and low in vitamins and minerals, could be involved in the higher prevalence of obesity [1,5]. 

Urbanization could be associated with increased sedentary behavior and lack of physical activity due to better access to cars and transportation. Urban families spend more time watching the television, using the internet and computer games and sleep less than rural families. Children’s diet from urban areas consists of fewer fruits and vegetables and a high amount of animal-based and high-energy food [11].

There is an agreement on the role of standards recommendation to define overweight and obesity in children and compare the prevalence in different populations [13]. Body mass index (BMI), a measure of weight for height, was used widely for analyzing obesity in children and adults worldwide and is the most practical, inexpensive and universally applicable [13]. 

Many reference systems define overweight and obesity in the children population. The WHO, IOTF and CDC references are the most used in studies, but these are based on different reference populations and cut-off values [14,15,16,17]. The cut-offs used in children and adolescents should be gender- and age-specific due to the BMI variability during childhood [18]. In WHO references, a BMI of 30 kg/m^2^ at the age of 18–19 years correspond to a 2SD from the median of the reference population (97.5^th^ percentile), while in IOTF reference a BMI of 30 kg/m^2^ at age 18 years corresponds to the 98.7^th^ percentile in girls and 98.9^th^ percentile in boys. There is no consensus on which reference set is the best one: IOTF and CDC references classified fewer children as overweight and obese than the WHO reference [18,19].

Overweight and obesity, but also underweight, are associated with health risks during the lifetime. Overweight in childhood is not only a precursor of obesity in adulthood but is also correlated with increased risk of chronic diseases, type 2 diabetes mellitus, cardiovascular diseases, musculoskeletal disorders, fatty liver (hepatic steatosis), cancers and psychosocial consequences (depression, low self-esteem, reduction of quality of life and a higher risk of bullying) [10,20]. The link between BMI and body fat and long-term cardiovascular risks of obesity in children was demonstrated. The cut-offs used to characterize overweight and obesity are good indicators of risks for adverse outcome [18]. Underweight is associated with the risk of infectious diseases, nutritional deficiencies, decreased cognitive development, and in girls at childbearing age, the risk for maternal mortality, preterm birth, intrauterine growth retardation or delivery complications [6,8].

Public health policies should emphasize the prevention of overweight and obesity starting at an early age and should be based on early diagnostic tools [2]. Health education for school-aged children is the most effective way to learn healthy lifestyle behavior and decrease the risk of obesity and overweight in adulthood [21]. There is a closed relationship between school education and obesity as education raises awareness of health and nutrition and helps children have healthier choices and lifestyles. However, the school-based campaign could not help decrease the prevalence of obesity without support from schools, families, communities and public health policymakers, who should cooperate for a better healthy environment for children [22].

Several studies evaluating overweight and obesity in children were published in the last decade for Romania, but there was no one national representative study [23,24,25,26,27,28,29]. The Health Behaviour for School-aged Children (HBSC) Study reported that for Romania in 2013–2014, the prevalence of overweight (including obesity) at the age of 11 years was 14% in girls and 33% in boys, and at the age of 13 years was 11% in girls and 26% in boys and at the age of 15 years was 10% in girls and 21% in boys [30].

This study aimed to estimate underweight, overweight and obesity prevalence in school-aged children in urban areas from the Western part of Romania using the WHO, CDC and IOTF references.

## 2. Materials and Methods

### 2.1. Population 

This cross-sectional study included a cohort of students enrolled in 177 public schools from five counties from the Western part of Transylvania, Romania (Cluj, Satu-Mare, Bihor, Arad, Timiș). The study was a part of the project “Development of skills of specialized medical staff on the prevention, diagnosis, and surgical treatment of congenital and acquired heart disease in children using state-of-the-art technology. CARDIOPED”. The schools chosen to be part of the study were those from the urban area of these counties, based on the data received from County School Inspectorates. We enrolled in this study 21,625 children, aged 7 to 18 years. 

The school physician sent the parents a letter with information and an invitation for their children to participate in the study. They were informed about the project and the data collected. All students aged 7 to 18 years, present at school on the days of data collection with the written consent of parents, were included in this study. Children without informed consent signed by their parents were omitted. Children with ages under 7 years and those with the age of 19 years and over were excluded from the analysis.

### 2.2. Data Collection

The study period was January and December 2016. The school physician, nurses and other staff performed the weight and height measurements during morning hours. All the personnel involved in weight and height measurements were trained to collect data correctly, based on WHO recommendations. The mean of three consecutive measurements was recorded. The body weight was measured using a standard scale to the nearest 0.1 kg. The height was measured to the closest 0.1 cm using a stadiometer. The body weight and height were measured with students standing erect, without shoes, only in underwear.

BMI was calculated based on the formula: BMI(kg/m^2^) = body weight(kg)/height^2^(m^2^).

To define underweight, overweight and obesity in children, we have used three criteria. The WHO Reference 2007, based on the 1977 National Center for Health Statistics (NCHS)/WHO reference, use the following cut-offs: underweight (thinness) <−2SD, overweight >+1SD (equivalent to BMI 25 kg/m^2^ at 19 years), obesity >+2SD (equivalent to BMI 30 kg/m^2^ at 19 years) [14,31].

Based on the National Center for Health Statistics and the CDC, BMI reference standards for children between 2 and 20 years of age, published in 2000, the cut-offs are the following: underweight <5^th^ percentile, overweight 85–95^th^ percentile, obesity >95^th^ percentile. Severe obesity was defined as BMI > 120% of the 95^th^ percentile [17].

The IOTF 2012 reference was based on national surveys from 6 countries (Brazil, Great Britain, Hong Kong, the Netherlands, Singapore, and the United States). IOTF BMI cut-off corresponds to a BMI of 18.5 kg/m^2^ (less than 15.5^th^ and 16.5^th^ percentile in boys and girls), 25 kg/m^2^ (greater than 90.5^th^ and 89.3^rd^ percentile in boys and girls) and 30 kg/m^2^ (greater than 98.9^th^ and 98.6^th^ percentile in boys and girls) at the age of 18 years defining underweight, overweight and obesity, respectively [15,16].

The study has been conducted according to the Declaration of Helsinki principles.

### 2.3. Data Analysis 

For each child, the WHO and CDC corresponding BMI z-score were calculated using the LMS method: z = ((BMI/M)^L-1)/LS, where the L, M, and S parameters were taken from the WHO (https://www.who.int/growthref/who2007_bmi_for_age/en/ (accessed on 30 April 2021)) and CDC (https://www.cdc.gov/growthcharts/percentile_data_files.htm (accessed on 30 April 2021)) reference tables, considering the sex and age of each child. The BMI z-score was then converted to percentile and compared to the WHO and CDC percentile reference to categorize each child as underweight, normal, overweight or obese.

Concerning the children’s classification according to IOTF, the calculated BMI was then compared with the IOTF BMI cut-off values according to the sex and age (https://www.worldobesity.org/about/about-obesity/obesity-classification).

The prevalence of each group was calculated. All analyses were performed using EXCEL 2019 (Microsoft Office).

## 3. Results

Our cohort consists of 21,625 children between the ages of 7 and 18, from the urban area of five counties from the Western part of Romania. There were 10,421 boys (48.19%) and 11,204 girls (51.81%). In Table 1, we present the distribution of our study population based on age and sex.

Using the WHO reference, the prevalence of overweight, obesity and severe obesity in our study population was 13.8%, 10.7% and 5.1%, respectively. The prevalence of underweight was 5.2%. In Table 2, Table 3 and Table 4, we present the prevalence of underweight, overweight, obesity and severe obesity for the study population, for boys and girls based on age.

Using the CDC reference, the prevalence of overweight, obesity and severe obesity in our study population was 16.2%, 10.0% and 1.2%, respectively. The prevalence of underweight was 6.0%. In Table 5, Table 6 and Table 7, we present the prevalence of underweight, overweight, obesity and severe obesity for the study population, for boys and girls based on age.

Based on the IOTF cut-offs, the prevalence of overweight, obesity and severe obesity in our study population was 20.3%, 5.7% and 1.6%, respectively. The prevalence of underweight was 2.6%. In Table 8, Table 9 and Table 10, we present the prevalence of underweight, overweight, obesity and severe obesity for the study population, for boys and girls based on age.

**Figure 1:**
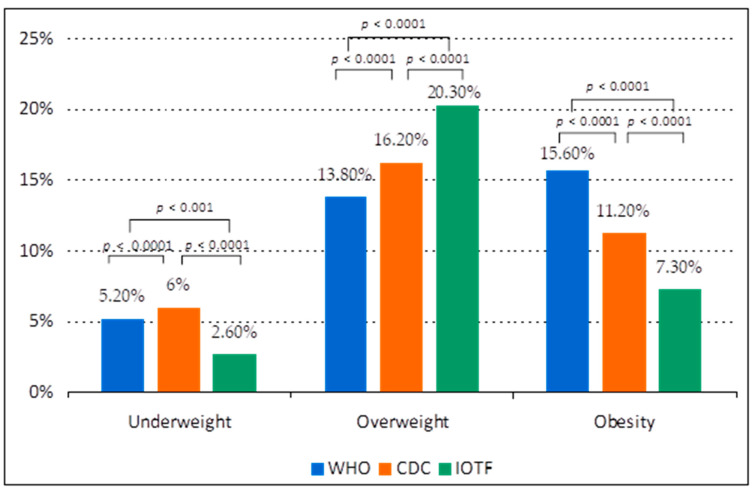
Prevalence of underweight, overweight and obesity in children aged 7–18 years using the WHO, CDC and IOTF references.

In our study, the prevalence of overweight (including obesity) in the school-aged population from the urban area was comparable using the three reference systems, 29.6% (WHO), 27.4% (CDC) and 27.6% (IOTF). The differences appear at the prevalence of overweight (higher using IOTF cut-offs, 20.3%), obesity (highest using WHO reference, 15.8%), and severe obesity (also highest based on WHO reference, 5.1%) (Figure 1). 

Prevalence of overweight (including obesity) was higher in boys (31.4%, 31.7%, 30.6%, based on WHO, CDC and IOTF respectively) than in girls (27.9%, 23.5%, 24.8%; *p* < 0.0001). Obesity (including severe obesity) had higher prevalence using WHO criteria (16.7% in boys and 14.9% in girls, *p* = 0.0003) than using CDC (14.2% in boys and 8.4% in girls, *p* < 0.0001), the lowest prevalence being when IOTF cut-offs were used (8.5% in boys and 6.2% in girls, *p* < 0.0001). 

The highest prevalence of overweight was found using WHO percentile cut-offs at 10 years of age (43.8% in boys and 37.8% in girls, *p* = 0.0056), and the lowest was at the age of 18 years (18.6% in boys and 11.1% in girls, *p* = 0.0043). Also, based on CDC and IOTF, the extremes values for the prevalence of overweight were at 10 years of age (42.5%, 38.5% in boys, 31.6%, 32.7% in girls; *p* < 0.0001 and *p* = 0.0060) and 18 years of age (14.4%, 18.7% in boys, 8.9%, 10.9% in girls; *p* = 0.0203 and *p* = 0.0029). If we analyze the obesity prevalence, we observed the same situation—the highest level at the age of 10 (except in girls, based on CDC and IOTF where the highest prevalence was at the age of 8) and the lowest level at the age of 18 years.

Our study showed a prevalence of severe obesity of 5.1% based on WHO criteria and lower ones, 1.2% and 1.6%, based on CDC and IOTF references, respectively. The severe obesity prevalence was higher in boys, based on CDC and IOTF cut-offs (1.8%, 1.8% in boys compared with 0.7%, 1.4% in girls; *p* < 0.0001 and *p* = 0.0189), but using WHO reference, the severe obesity was almost similar in both sexes (*p* = 0.5043). Based on the WHO definition, the highest prevalence of severe obesity was found in children aged 7–10 years, with the highest level in girls at 8 years (10.3%). CDC and IOTF criteria for severe obesity revealed the highest prevalence in both sexes at 7–8 years. The lowest prevalence for severe obesity was in children aged 17–18 years. 

The underweight prevalence was higher using WHO and CDC reference (5.2% and 6%) than IOTF cut-offs (2.6%). Based on CDC criteria, more boys were classified as underweight than girls (6.2% vs. 5.8%, *p* = 0.2156) but using WHO and IOTF criteria, the prevalence of underweight was higher in girls (5.6% vs. 4.8%, *p* = 0.0082 and 3.1% vs. 2%, *p* < 0.0001). The underweight prevalence was higher in lower ages (the highest prevalence was in boys aged 7, using CDC criteria, 10.6%) and lower in 17–18 years old children (based on WHO standards) and in children aged 14 years (based on CDC and IOTF criteria). Using CDC references, the girls aged 18 years had the highest prevalence of underweight of 9.4%.

## 4. Discussion

As a part of a larger project on cardiovascular diseases in children, this study aimed to analyze the prevalence of overweight and obesity in school-aged children population from the urban area in the northwestern part of Romania. We also studied the underweight in children as this continues to be an issue in developing countries.

During the last three decades, many demographic, epidemiological and socioeconomic changes took place in Romania as in other countries from Eastern Europe. The improvement of socioeconomic status in most developing countries came with better access to healthcare and less physical activity and a nutritional transition to a high intake of carbohydrates and saturated fat due to highly processed food and fast food. These changes were seen in all Eastern European countries and were associated with a sharp increase in overweight and obesity in children [24,27,32]. This trend should be followed by implementing strategies to fight against childhood obesity and its risk for future adult health. 

The prevalence of overweight (including obesity) in our study population based on WHO, CDC and IOTF references was almost similar, 29.6%, 27.4% and 27.6%, respectively, but with differences between the overweight and obesity classification using these references. A higher prevalence of overweight in boys was found in almost all other studies [11,19,32,33]. Regarding age, the trend was similar no matter the reference used: excess weight prevalence was highest in children before puberty (up to 10–12 years) and lowest in adolescents aged 17–18 years. This decreasing prevalence of overweight/obesity from the pre-puberty period to 18 years was reported in other studies from Romania or other countries [25,34,35].

### 4.1. Prevalence of Overweight and Obesity in Other Countries 

There are essential differences in the prevalence of overweight and obesity in children in European countries, from the lowest level in Slovakia (9%), Turkey and the Netherlands (10.5%) to the highest levels in Italy (26%) and the United Kingdom (29%) [36]. Worldwide, it varies from almost 1 in 3 children in the U.S. [37], Australia (increasing from 24.6% in 2007/2008 to 27.6% in 2014/2015, [38]) and New Zealand (33%, [39]) to lower prevalence of overweight (14.7%) and obesity (5.8%) in Eastern Ethiopia in 2016 [40]. Table 11 presents the prevalence of overweight (including obesity) and obesity in children in different countries, based on various criteria.

In Germany, The Second Wave of the German Health Interview and Examination Survey for Children and Adolescents (KiGGS Wave 2, 2014–2017) showed that the overall prevalence in children aged 3 to 17 years for overweight was 15.4% and for obesity was 5.9%, with no difference between boys and girls [20]. This study revealed that the obesity prevalence increased from 3–6 years (3.2% in girls and 1% in boys) to 14–17 years (7.7% in girls and 9.2% in boys). There was no increase in prevalence since 2003–2006 [20].

The increase of the overweight (including obesity) with age was also reported in the U.S., from 22.8% at age 2–5 years, 34.2% at the age of 6–11 years and 34.5% in adolescence (12–19 years). The same trend was reported for obesity, from 8.4% in pre-school children, 17.7% at the age of 6–11 years and 20.5% in adolescence [37]. In the studies coming from the U.S. [41], Portugal [33], Spain [19], Ukraine [32], boys, in all age groups, had a higher prevalence of obesity than girls. Still, other studies reported higher prevalence in girls, in France [2] or Spain [42], or even no differences based on sex, as in Serbia [36] or Germany [20]. In Asian countries, the prevalence of overweight and obesity increased with age and was higher in boys than girls. [11] Compared to the U.S. or Asian countries, the prevalence of overweight decreased in adolescence in our study.

One systematic review of 32 studies published between 2006–2016 with data from 27 European countries revealed that the pooled prevalence estimate of overweight was 14.6% and obesity was 5.3% in pre-school children (aged 2–7 years). All studies used the IOTF definition criteria. There is significant heterogeneity in Europe, but Southern Europe (countries like Italy and Greece) had the highest prevalence of overweight. Compared with our results, in pre-school children in most European countries, girls showed a higher prevalence of overweight or obesity than boys [45].

An analysis performed in primary school children, including 636,933 children from 21 countries participating in the first three COSI (The WHO European Childhood Obesity Surveillance Initiative), revealed significant differences among countries with higher values of the prevalence of severe obesity in Southern Europe (Greece, Malta, Italy, Spain and San Marino), above 4%. Based on the COSI study, Romania is in the middle group of the European countries, with a prevalence of severe obesity of 2.2% using the WHO definition (3.8% in boys and 0.5% in girls) and 1.2% using the IOTF definition (1.7% in boys and 0.6% in girls) [46]. Our data for children 7–10 years old shows a higher prevalence of severe obesity using CDC and IOTF criteria.

### 4.2. Other Studies Analyzing Overweight and Obesity in Romanian Children 

During the last years, there was no national representative study on the prevalence of overweight and obesity in Romania, but studies from different regions of the country. In the western part of the country, the prevalence of overweight (including obesity) and obesity in children aged 7–18 years was 25.4% and 7.2%, while in the urban area in the northeastern part in children aged 6–10 years, it was 23.7% and 7.1%, using IOTF criteria [23,24]. In a study published in 2009, the prevalence of overweight (including obesity) and obesity in school children from Cluj-Napoca, using IOTF criteria, was 21.1% and 8.3%. The prevalence decreased from the age group of 6–10 years to adolescents [25]. In Bucharest, the capital city of Romania, the prevalence of overweight (including obesity) and obesity were similar with the other studies from the country: 31.6% and 11.4% (WHO), 24.6% and 6.2% (IOTF), 25.2% and 10% (CDC). This study tried to analyze the eating behaviors, with almost all participants reporting at least one unhealthy eating behavior, but there was no significant relationship found with overweight or obesity [27]. In a study performed in Bihor county, in the Western part of Romania, published in 2016, in school-aged children between 11–15 years, 15.3% were overweight and 12% obese. Overweight and obesity were more prevalent in children from the urban area (30%) than in rural areas (24%) [26]. In all these studies [23,24,25,26,27], the prevalence of overweight and obesity was higher in boys than in girls, similar to our results.

In a pooled analysis of cross-sectional studies published between 2006 and 2015, including 25,060 children aged 6–19 from 8 counties from different regions of Romania, the prevalence of overweight (including obesity) was 28.3%/23%/23.2% (WHO/IOTF/CDC). The prevalence was smaller than in our study, probably due to the inclusion of urban and rural populations in the pool of studies [29]. Even lower prevalence was reported in 2017 in school-aged children: overweight decreased from 21.28% in boys and 13.89% in girls at 14 years old to 15.20% in boys and 5.86% in girls at 18 years. The obesity prevalence varied in girls from 1.85% at 14 years to 0.85% at 16 years and in boys between 2.12% at 15 years to 3.55% at 17 years [34,35]. Compared to the results presented for Romania by Cattaneo [47], in 2004, all studies performed after 2010, including our research, revealed a higher prevalence of overweight and obesity in school-aged children, with a decrease in adolescence. 

### 4.3. The Role of Urbanization in the Prevalence of Overweight and Obesity

Differences in the prevalence of excess weight in children by demographics were reported in different countries. In the U.S., the prevalence of overweight was different based on the level of urbanization [48]. In contrast, in a cross-sectional analysis including children aged 2–19 years, differences in obesity by urbanization level were not significant (21.7% vs. 17.1%). Moreover, severe obesity was more prevalent in non-metropolitan areas than in large metropolitan areas (9.4% vs. 5.1%). Analyzing the trend, the prevalence of obesity increased in large urban areas from 15.3% in 2001–2004 to 17.1% in 2013–2016, and in non-metropolitan areas from 20% to 21.7%. The severe obesity prevalence increased from 4.3% to 5.1% in large metropolitan areas and 7.3% to 9.4% in non-metropolitan areas [49]. In Florida, in a study based on data from 2017, the highest prevalence of obesity and severe obesity was observed in rural-small town areas, higher with 25% and 75%, respectively, compared to metropolitan areas. These data contradict studies from other countries, but the data could be biased as children from low-income families and without insurance may have been underrepresented in the sample [41].

A higher prevalence of overweight in urban areas was reported in developing countries but attenuated after adjusting for the socioeconomic status [50]. In a systematic review of data from Asian countries in the urban population, the prevalence of overweight in children aged 5–11 years was 12.6% in boys and 10.8% in girls. For adolescents, it was 17.1% in boys and 14.8% in girls. The prevalence of obesity in the same urban population was for children 6.6% in boys and 4.6% in girls and for adolescents 10.3% in boys and 6.6% in girls. The Asian studies reported a nutrition transition and urbanization in most of Asia associated with a sedentary lifestyle and dietary changes [11]. In Europe, in studies from Slovakia and Poland, the excess weight is more prevalent in urban areas than in rural ones [51,52]. Conversely, in Greece, there was no significant difference [43].

In Romania, the COSI study presented a higher prevalence of overweight in the urban area (31.4%) than in the rural area (21.6%) [53]. Also, in the pooled analysis of Romania’s studies, the excess weight was more prevalent in the urban area (29.5%) than in rural areas (22.9%) [29]. In our study, the prevalence of overweight in the urban population was lower than in the COSI study.

### 4.4. Comparison of Different References for Overweight and Obesity Definition 

It is challenging to choose the most appropriate reference system for overweight and obesity classification. Depending on the aim, IOTF reference should be used for international comparison, and the other references for clinical use [54].

The WHO and IOTF references produced a similar overall prevalence of overweight and obesity in a sample population from China, Russia and the U.S. [18]. In developing countries, the prevalence of childhood obesity was higher than adolescent obesity compared to developed countries as children mature later than in countries used as reference populations for WHO and IOTF systems [18]. Population-specific standards should be used in different countries as there could be differences in BMI-body fat and BMI-risk for disease relationships. IOTF and WHO references are statistically defined and not in a relationship with evidence of long-term health risks [18].

In a French study, the WHO system was significantly different from IOTF, as a higher proportion of children were considered overweight (20% vs. 16.2%) and obese (11.6% vs. 6.7%) [2]. Similarly, in studies coming from Portugal and Spain, the WHO cut-offs estimate the highest prevalence of overweight, obesity and severe obesity, and IOTF criteria the smallest. The best agreement for overweight and obesity evaluation was between CDC and IOTF cut-offs [13,19,33,55]. 

WHO and CDC references based on the U.S. population seem not to be valid in other populations from Asia, Africa or other developing countries. IOTF references, based on a sample population from six countries (U.S., Brazil, Great Britain, Hong Kong, The Netherlands, Singapore), are closer to developed countries and the Western population than to other countries. When we compare the results of obesity prevalence based on different references and populations (sampling, age), we should be aware of those kinds of limitations [11,18]. If the results based on WHO references would be the basis for tailoring public health measures, some actions will target normal-weight children. In many countries, using IOTF references would be more appropriate for evaluating overweight and obesity prevalence as this system is the best adapted for that population [2].

Our study also results in a different prevalence of underweight, overweight, obesity and severe obesity based on the criteria used for analysis. In Romania, the last growth charts were published in 1974. As there are questions as to whether WHO standards are helpful for the Romanian child population, in 2016, synthetic growth references were developed. The authors used data from six studies with anthropometric information obtained between 1999 and 2016. If comparing this synthetic reference with WHO and CDC references, there are statistically significant differences. Romanian children are taller than the WHO and CDC reference. The new reference shows that Romanian children may have a higher BMI than both WHO and IOTF references. There were some limitations of this reference, one being the fact that it was created mainly from studies consisting of children from urban areas [28]. Based on all these aspects, we can assume that the IOTF may be the most useful also for our country. The economic and epidemiological changes during the last 20 years bring our population closer to developed countries and the Western population than to other countries.

### 4.5. Underweight in Children and Adolescents

Underweight continues to be a problem in some countries, and together with overweight consists of a double burden for public health authorities. Pediatricians should have an essential role in advocating for healthy child nutrition programs to decrease malnutrition prevalence due to food insecurity [56]. 

In Eastern Europe, 15.2% of children aged 6–19 years were underweight in Ukraine [32] and 24.2% in Poland, in Podalskie province [57]. Another study from Poland found the highest prevalence of underweight in girls at the age of 13 (19.3%) and in boys at the age of 14 (8.7%) [58]. Still, more pre-school-aged children are underweight (10.4%) than overweight (6.4%) [59]. In the northwestern part of Russia, in a rural area, the prevalence of stunting was 3.3%, 5.2%, 4.5%, and underweight was 3.6%, 3.1%, 2.3% (based on the WHO-2007, CDC-2000, and Russian criteria). Using the IOTF definition, the underweight prevalence was only 1.8% [44]. A recently published meta-analysis, including 323,420 European children aged 2 to 18 years, revealed an increasing trend in Eastern, Northern and Southern Europe and descending trend in Western Europe, with the highest prevalence in Eastern (12%) and Western Europe (11.8%) [8]. In our study, the prevalence of underweight was 5.2%, 6.0% and 2.6% using WHO, CDC and IOTF criteria, and lower than other countries from Eastern Europe [8,9]. Again, using different criteria, the prevalence of underweight is quite different in the same population, giving us the possibility to conclude that we need to choose appropriate standards. 

Underweight is frequently overlooked by health policymakers from developed countries. Future interventions should increase the awareness of underweight as population strategies for reducing obesity may harm malnourished children [8].

### 4.6. Limitations of the Study

The cross-sectional design of the study, including data from 2016, did not allow us to analyze the trend of obesity and overweight in our children. Even though the main project included a questionnaire on lifestyle, we could not correlate with nutrition, feeding behavior and sedentary habits due to methodological issues. The study population was enrolled in schools from the urban area, but there was no analysis of the students’ living place. In some schools of metropolitan regions, children living in the surrounding rural area could be enrolled. Due to some lack of collaboration, we could not include all schools from the five counties’ urban areas. There was no possibility of comparing the prevalence of overweight and obesity with school-aged children from the rural area due to the main project’s methodological limitation. Besides all these limitations, the large number of children enrolled in the study using the same method make this study the most extensive single study analyzing the prevalence of underweight, overweight and obesity in a large region of Romania.

## 5. Conclusions

The prevalence of overweight (including obesity) in children at school-age from the urban area of Western Romania is estimated at under 30%. The highest prevalence of overweight was at pre-puberty age, with a decrease toward the age of 18. Boys were with a higher prevalence of overweight/obesity than girls. The WHO criteria resulted in the highest prevalence of obesity and IOTF cut-offs in the lowest. Based on WHO, 5.1% of children are with severe obesity, but the other references revealed a lower prevalence (1.2% and 1.6%). Underweight is still an essential issue for developing countries and had a low prevalence in children from urban areas (5.2%, 6%, 2.6%, based on WHO, CDC, and IOTF criteria). It is essential to choose the most appropriate reference system to define overweight and obesity if one wants to correctly evaluate public health measures to halt obesity in children.

## Figures and Tables

**Table 1:** Study population: distribution based on age and sex.

Age (Years)	Boys *n* (%)	Girls *n* (%)	Total
7	585 (49.45%)	598 (50.55%)	1183
8	886 (52.00%)	818 (48.00%)	1704
9	845 (47.47%)	935 (52.53%)	1780
10	1015 (49.32%)	1043 (50.68%)	2058
11	1141 (49.63%)	1158 (50.37%)	2299
12	999 (48.97%)	1041 (51.03%)	2040
13	982 (47.88%)	1069 (52.12%)	2051
14	989 (47.70%)	1084 (52.30%)	2073
15	1017 (45.93%)	1197 (54.07%)	2214
16	921 (47.82%)	1005 (52.18%)	1926
17	709 (45.10%)	863 (54.90%)	1572
18	332 (45.79%)	393 (54.21%)	725
Total	10,421 (48.19%)	11,204 (51.81%)	21,625

**Table 2:** Prevalence of underweight, overweight, obesity and severe obesity using WHO percentile.

WHO	Underweight	Normal	Overweight	Obesity	Severe Obesity	Total
BMI Percentile	<5	5–85	85–95	95–99	≥99	
Number	1117	14,105	2989	2309	1105	21,625
Prevalence	5.2%	65.2%	13.8%	10.7%	5.1%	100.0%

**Table 3:** Underweight, overweight and obesity prevalence in boys, according to age, using WHO percentile reference.

WHO, Boys Age (Years)	Underweight	Normal	Overweight	Obesity	Severe Obesity
7	4.4%	65.5%	12.3%	10.6%	7.2%
8	5.3%	59.8%	13.3%	13.1%	8.5%
9	5.0%	58.6%	13.1%	15.6%	7.7%
10	4.4%	51.7%	16.9%	18.3%	8.6%
11	4.6%	61.3%	16.9%	12.4%	4.8%
12	5.8%	58.5%	16.9%	14.0%	4.8%
13	4.9%	64.3%	17.2%	11.1%	2.5%
14	4.2%	67.6%	14.6%	9.7%	3.7%
15	5.5%	69.6%	13.6%	8.1%	3.2%
16	4.9%	68.9%	14.0%	9.6%	2.6%
17	3.8%	74.2%	11.4%	7.3%	3.2%
18	2.1%	79.2%	11.1%	5.7%	1.8%
Total	4.8%	63.8%	14.7%	11.7%	5.0%

**Table 4:** Underweight, overweight and obesity prevalence in girls, according to age, using WHO percentile reference.

WHO, Girls Age (Years)	Underweight	Normal	Overweight	Obesity	Severe Obesity
7	5.2%	63.7%	12.7%	11.0%	7.4%
8	7.1%	59.4%	12.3%	10.9%	10.3%
9	6.1%	58.4%	14.8%	14.4%	6.3%
10	6.6%	55.6%	16.1%	14.6%	7.1%
11	7.3%	59.8%	15.3%	11.9%	5.7%
12	6.1%	61.6%	15.8%	10.7%	6.0%
13	4.9%	65.4%	13.8%	11.5%	4.5%
14	4.6%	69.7%	13.6%	8.1%	4.0%
15	5.2%	75.3%	10.7%	5.6%	3.3%
16	4.3%	75.4%	10.8%	5.2%	4.3%
17	3.8%	79.5%	8.5%	6.3%	2.0%
18	4.8%	84.0%	7.1%	2.5%	1.5%
Total	5.6%	66.5%	13.0%	9.7%	5.2%

**Table 5:** Prevalence of underweight, overweight, obesity and severe obesity using CDC percentile.

CDC	Underweight	Normal	Overweight	Obesity	Severe Obesity	Total
BMI percentile	<5	5–85	85–95	95–99	≥99	
Number	1295	14,393	3512	2156	269	21,625
Prevalence	6.0%	66.6%	16.2%	10.0%	1.2%	100.0%

**Table 6:** Underweight, overweight and obesity prevalence in boys, according to the age, using CDC percentile reference.

CDC, Boys Age (Years)	Underweight	Normal	Overweight	Obesity	Severe Obesity
7	10.6%	59.3%	14.9%	11.5%	3.8%
8	9.8%	57.0%	15.6%	14.2%	3.4%
9	7.3%	56.6%	17.6%	16.4%	2.0%
10	6.1%	51.4%	21.1%	19.4%	2.0%
11	5.0%	61.8%	19.3%	13.1%	0.9%
12	5.8%	58.4%	20.0%	14.7%	1.1%
13	4.1%	63.6%	20.0%	11.3%	1.0%
14	3.5%	65.2%	19.1%	10.0%	2.1%
15	5.2%	67.0%	16.7%	9.0%	2.1%
16	5.8%	67.4%	15.4%	10.3%	1.1%
17	6.8%	72.4%	12.4%	6.6%	1.8%
18	8.1%	77.4%	7.8%	5.7%	0.9%
Total	6.2%	62.2%	17.5%	12.4%	1.8%

**Table 7:** Underweight, overweight and obesity prevalence in girls, according to the age, using CDC percentile reference.

CDC, Girls Age (Years)	Underweight	Normal	Overweight	Obesity	Severe Obesity
7	7.2%	63.9%	16.1%	10.0%	2.8%
8	9.3%	60.6%	16.9%	11.2%	2.2%
9	7.6%	63.0%	19.0%	9.2%	1.2%
10	7.6%	60.9%	19.9%	10.6%	1.1%
11	7.3%	67.3%	16.6%	8.5%	0.3%
12	5.4%	68.6%	16.5%	8.7%	0.8%
13	3.4%	72.2%	15.8%	8.2%	0.4%
14	3.3%	73.2%	15.9%	7.1%	0.5%
15	3.8%	79.2%	11.5%	5.2%	0.3%
16	4.2%	78.2%	11.3%	6.0%	0.3%
17	5.2%	80.4%	10.7%	3.7%	0.0%
18	9.4%	81.7%	6.4%	2.5%	0.0%
Total	5.8%	70.6%	15.1%	7.7%	0.7%

**Table 8:** Prevalence of underweight, overweight, obesity and severe obesity using IOTF cut-offs.

IOTF	Underweight	Normal	Overweight	Obesity	Severe Obesity	Total
BMI cut-off	<17	17–25	25–30	30–35	≥35	
Number	562	15,100	4380	1242	341	21,625
Prevalence	2.6%	69.8%	20.3%	5.7%	1.6%	100.0%

**Table 9:** Underweight, overweight and obesity prevalence in boys, according to the age, using IOTF cut-offs.

IOTF, Boys Age (Years)	Underweight	Normal	Overweight	Obesity	Severe Obesity
7	4.4%	69.9%	17.4%	5.1%	3.1%
8	4.1%	67.3%	19.3%	6.0%	3.4%
9	3.1%	64.6%	23.1%	6.9%	2.4%
10	1.7%	59.8%	25.9%	10.5%	2.1%
11	1.8%	67.5%	24.1%	5.8%	0.9%
12	1.6%	63.3%	26.0%	7.5%	1.6%
13	1.0%	67.2%	24.2%	6.5%	1.0%
14	0.9%	67.4%	23.0%	6.7%	2.0%
15	1.4%	69.9%	20.0%	6.8%	2.0%
16	2.1%	68.7%	21.5%	6.6%	1.1%
17	1.7%	73.9%	17.5%	5.6%	1.3%
18	1.5%	79.8%	13.9%	3.9%	0.9%
Total	2.0%	67.4%	22.1%	6.7%	1.8%

**Table 10:** Underweight, overweight and obesity prevalence in girls, according to the age, using IOTF cut-offs.

IOTF, Girls Age (Years)	Underweight	Normal	Overweight	Obesity	Severe Obesity
7	3.8%	67.4%	19.2%	5.9%	3.7%
8	4.8%	65.0%	19.2%	8.2%	2.8%
9	3.9%	65.9%	23.4%	5.2%	1.6%
10	3.9%	63.3%	25.0%	6.0%	1.7%
11	3.5%	69.2%	21.4%	5.6%	0.3%
12	2.8%	69.6%	20.9%	5.8%	0.9%
13	1.9%	73.2%	19.0%	4.7%	1.3%
14	2.3%	73.9%	18.7%	4.2%	0.9%
15	2.3%	79.7%	13.4%	3.1%	1.4%
16	2.8%	77.1%	15.0%	3.7%	1.4%
17	3.1%	80.5%	12.6%	2.9%	0.8%
18	3.8%	85.2%	8.4%	2.0%	0.5%
Total	3.1%	72.1%	18.6%	4.8%	1.4%

**Table 11:** Prevalence of overweight (including obesity) and obesity in children in different countries, based on various criteria.

Country, Year, Reference Number	Overweight, Including Obesity (%)		Obesity (%)		Age (years)	Reference Criteria
	Boys	Girls	Boys	Girls		
Romania, 2016	31.4%	27.9%	16.7%	14.9%	7–18	WHO
	31.7%	23.5%	14.2%	8.9%		CDC
	30.6%	24.8%	8.5%	6.2%		IOTF
France, 2011, [2]	14.0%	19%	2.5%	4%	7–11	IOTF
Portugal, 2014, [33]	31.6%	20.7%	10.9%	5.4%	10–16	WHO
	25.2%	12.8%	9.4%	4.4%		CDC
	23.9%	13.3%	5.4%	2%		IOTF
Spain, 2011, [19]	44%	36%	19%	16%	2–15	WHO
	32%	30%	11%	12%		IOTF
Germany, 2017, [20]	15.6%	15.3%	6.3%	5.5%	3–17	German
Greece, 2011, [43]	29.9%	29.2%	12.9%	10.6%	10–12	IOTF
Ukraine, 2018, [32]	21.8%	13.8%	5.8%	2.7%	6–18	WHO
	15.5%	9.9%	4.8%	2.5%		CDC
	13.8%	8.5%	2.3%	1.8%		IOTF
Serbia, 2011, [36]	10%		5%		15–18	CDC
Russia, 2010, [44]	10.3%		4.7%		14–17	WHO
	8.6%		2.6%			CDC
	8.6%		2.3%			Russian
	9%		2%			IOTF
United States, 2011, [37]	22.8%		8.4%		2–5	CDC
	34.2%		17.7%		6–11	
	34.5%		20.5%		12–19	
Asia, 2018, [11] Pool of 41 studies	18.7%	15.7%	7%	4.8%	5–11	WHO/IOTF
	26.0%	19.9%	10.1%	6.2%	12–19	

WHO, World Health Organization, IOTF, International Obesity Task Force, CDC, Center for Disease Control and Prevention.

## Data Availability

The data presented in this study are available on request from the corresponding author.
